# A survey of core and support activities of communicable disease surveillance systems at operating-level CDCs in China

**DOI:** 10.1186/1471-2458-10-704

**Published:** 2010-11-17

**Authors:** Weiyi Xiong, Jun Lv, Liming Li

**Affiliations:** 1 Department of Epidemiology and Biostatistics, School of Public Health, Peking University Health Science Center, 38 Xueyuan Road, Beijing 100191, China

## Abstract

**Background:**

In recent years, problems like insufficient coordination, low efficiency, and heavy working load in national communicable disease surveillance systems in China have been pointed out by many researchers. To strengthen the national communicable disease surveillance systems becomes an immediate concern. Since the World Health Organization has recommended that a structured approach to strengthen national communicable disease surveillance must include an evaluation to existing systems which usually begins with a systematic description, we conducted the first survey for communicable disease surveillance systems in China, in order to understand the situation of core and support surveillance activities at province-level and county-level centers for disease control and prevention (CDCs).

**Methods:**

A nationwide survey was conducted by mail between May and October 2006 to investigate the implementation of core and support activities of the Notifiable Disease Reporting System (NDRS) and disease-specific surveillance systems in all of the 31 province-level and selected 14 county-level CDCs in Mainland China The comments on the performance of communicable disease surveillance systems were also collected from the directors of CDCs in this survey.

**Results:**

The core activities of NDRS such as confirmation, reporting and analysis and some support activities such as supervision and staff training were found sufficient in both province-level and county-level surveyed CDCs, but other support activities including information feedback, equipment and financial support need to be strengthened in most of the investigated CDCs. A total of 47 communicable diseases or syndromes were under surveillance at province level, and 20 diseases or syndromes at county level. The activities among different disease-specific surveillance systems varied widely. Acute flaccid paralysis (AFP), measles and tuberculosis (TB) surveillance systems got relatively high recognition both at province level and county level.

**Conclusions:**

China has already established a national communicable disease surveillance framework that combines NDRS and disease-specific surveillance systems. The core and support activities of NDRS were found sufficient, while the implementation of those activities varied among different disease-specific surveillance systems.

## Background

Surveillance is the ongoing systematic collection, analysis, and interpretation of outcome-specific data for use in planning, implementing and evaluating public health policies and practices [1]. It is generally accepted that communicable disease surveillance is the cornerstone of communicable disease prevention and control [2-4]. In People' Republic of China, since the establishment of the main communicable disease surveillance system, the Notifiable Disease Reporting System (NDRS), in 1950 s, many disease-specific surveillance systems had been developed as important complements to NDRS [5-7]. In 2004, the internet-based real-time information reporting technique was integrated into NDRS to facilitate the case-based report, which involved all the regional centers for disease control and prevention (CDCs) and more than 80% of public hospitals in China [8]. A total of 39 diseases are currently covered by NDRS, and the completeness and timeliness of case report have been dramatically improved since 2004. In 2005, based on previous works, Chinese Center for Disease Control and Prevention (CCDC) established the Enhanced Infectious Diseases and Vectors Surveillance Systems with the target of 25 diseases and 4 important vectors, and set up hundreds of surveillance sites throughout the country [9]. These systems generated a large amount of data on patient information, pathogens, and risk factors. These data have provided the scientific evidence for decision-making in the communicable diseases prevention and control in China.

**Figure 1:**
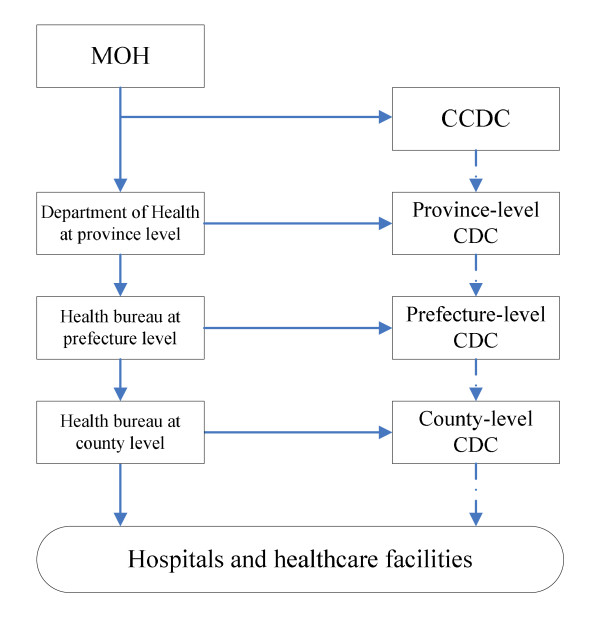
**Organization chart of communicable disease surveillance system in China**. The real line means playing the administrative management role; the dot line means playing the technical guidance role.

With all those achievements, there are some challenges need to be addressed. As a very large country with 31 provinces, municipalities and autonomous regions in the Mainland, the spectrum of diseases and environmental and socioeconomic conditions vary widely among different regions in China, so do the performance of communicable disease surveillance systems. The surveillance activities in China are mainly performed in three administrative levels: central level, province level, and county level (Figure 1) [10]. At central level CCDC, a subordinate department of the Ministry of Health (MOH), takes the responsibility for management of communicable disease surveillance systems. The province-level CDCs, one in each province, are in charge of management of communicable disease surveillance systems in their assigned territories. The county-level CDCs, also one in each county, are the primary public health units that monitor the epidemic of communicable diseases, investigates outbreaks, and coordinates all the hospitals, healthcare centers, clinics and laboratories to report communicable disease cases in their counties. NDRS is centrally planned and supported by all levels of the governments. Most of the disease-specific surveillance systems are also centrally planned, but operated under vertical management structures and their performance can vary greatly across different systems. The data are seldom shared across vertical programs. Furthermore, public health staff, in particular those working at the county-level CDCs, may have heavy workloads as they are involved in multiple systems which using different methodologies, terminologies, and reporting forms.

In recent years, the problems mentioned above such as insufficient coordination, low efficiency, and overloaded working burden were pointed out by many researchers [11-14]. They were considered to be ball and chain around the neck of national communicable disease surveillance and response systems in China. Consequently it becomes an immediate concern to strengthen the national communicable disease surveillance systems.

As WHO recommended, a structured approach to strengthen national communicable disease surveillance systems must include evaluation of existing systems to review strengths, weaknesses, and opportunities for improvement, and the components of surveillance system for evaluating comprise the public health importance of the targeted disease, the structure, the functions, and the attributes of the system [15]. Such evaluations had been conducted in China, but most of which were focused on the structure and attribute of single system. To date, there is no publicly disclosed comprehensive evaluation or systematic description of national communicable disease surveillance systems in China. The purpose of this paper was to provide a comprehensive profile for national communicable disease surveillance systems in China. Previous studies by WHO and US CDC [16-19] usually described the entire national communicable disease surveillance and response systems which covered all the involved organizations from health administrative departments to hospitals, this study were more concerned about how the existing surveillance systems worked at the operating level, i.e. province-level and county-level CDCs in China.

## Methods

### Definition and survey tool

The definition for core and support activities of communicable disease surveillance in China was developed by following the conceptual framework of communicable disease surveillance proposed by US CDC and WHO [20-22] (table 1). Given that this study was focused on province-level and county-level CDCs, those activities mainly conducted by hospitals, health administrative departments and CCDC were excluded.

**Table 1:** Core and support activities as tasked by communicable disease surveillance system in China

CDCs	Core activities					Support activities			
		
	Detection	Confirmation	Reporting	Analysis	Feedback	Training	Supervision	Equipment Support	Financial support
Province-level	-	√*	√	√	√	√	√	√	√
County-level	√*	√	√	√	√	√	√	√	√

* When necessary.

Definitions of activities in table 1 are in accordance with that proposed by McNabb SJ et al in 2002 [20]. The activity of resource-provision was divided into two: equipment and financial support.

Several questionnaires (additional file 1, 2, 3, 4, 5, 6)were designed by following an assessment tool recommended by WHO [22], to investigate whether the core and support activities were performed by NDRS and disease-specific surveillance systems at different level CDCs. The comments on the performance of surveillance systems were simultaneously collected from the directors of CDCs.

A pilot study was conducted in two province-level CDCs and two county-level CDCs. The respondents of pilot study were requested to answer two questions after finishing the questionnaire: Has the questionnaire covered all the main areas of existing surveillance activities? Which item is the most time-consuming? Final questionnaires were adjusted corresponding to the results of pilot study. Then a nationwide survey was conducted by mailing questionnaires to all the province-level CDCs and selected county-level CDCs.

### Sampling

All of the 31 province-level CDCs in Mainland China were investigated.

From nearly 3000 county-level CDCs across China, 14 were selected as a typical sample by a two-step framework. Firstly, all the 31 province-level CDCs were divided into 3 groups according to their scores on the quality of notifiable disease case report in year 2004 from an annually updated analysis report of NDRS by CCDC, which is the only national document evaluating the communicable disease surveillance system in China. The upper quartile of the provinces with the best reporting quality are Group A, the middle fifty percent are Group B and provinces in the lower quartile are Group C. Two to four provinces were sampled from each group based on their socioeconomic status. Finally, 8 provinces (Tianjin, Zhejiang, Gansu, Hubei, Hebei, Guizhou, Yunnan and Anhui) were selected for further sampling. Secondly, the chief directors of the selected province-level CDCs were requested to nominate two counties in their jurisdiction, one representing the good county-level performance of communicable disease surveillance and the other one representing the weak performance. Finally 14 county-level CDCs were chosen for this study (Gansu and Hebei each nominated 1 county of good performance for pilot study).

### Statistical Analysis

Epidata 3.0 was used for data entry. After data cleansing, the quality of returned questionnaire was evaluated. The questionnaire would be excluded from analysis if there are more than 5 questions missing. For each systems investigated, the proportion of the CDCs that have had the activities among all the same level CDCs was calculated.

## Results

A total of 31 province-level and 14 county-level CDCs were investigated between May and October 2006. All the respondents were asked to describe the related surveillance works performed in the previous calendar year in accordance with the questionnaires. 91.9% of the province-level and 100% of the county-level CDCs returned completed questionnaires. Quality evaluation showed that the proportions of invalid questionnaire in both province-level and county-level CDCs were acceptable (6.9% and 0.1% respectively).

### The activities of NDRS

The directors of the NDRS administrative branches from 28 province-level and 14 county-level CDCs responded to this survey. Both province-level and county-level CDCs had already taken the main core activities such as confirmation, reporting, and analysis, so did some support activities such as supervision and training. However some activities including feedback, equipment and financial supports, especially overtime pay provision, were found insufficient in most of the CDCs. (table 2 and 3)

**Table 2:** Core activities of NDRS in province-level and county-level CDCs in China

Core activities	Indicators	At province level	At county level
Confirmation	Proportion of CDCs having verified NDRS report from medical facilities in last calendar year	NA	14/14
Analysis	Proportion of CDCs having analyzed NDRS data on a regular basis in last calendar year	28/28	14/14
Report	Proportion of CDCs having submitted NDRS report to county-level health administrative departments in last calendar year	NA	14/14
& Feedback	Proportion of CDCs having received feedbacks from county-level health administrative departments in last calendar year	NA	4/14
	Proportion of CDCs having submitted NDRS report to prefecture-level health administrative departments in last calendar year	NA	4/14
	Proportion of CDCs having received feedback from prefecture-level health administrative departments in last calendar year	NA	1/4
	Proportion of CDCs having submitted NDRS report to province-level health administrative departments in last calendar year	28/28	NA
	Proportion of CDCs having received feedback from province-level health administrative departments in last calendar year	14/28	NA
	Proportion of CDCs having submitted NDRS report to MOH in last calendar year	3/28	NA
	Proportion of CDCs having received feedback from MOH in last calendar year	0/28	NA
	Proportion of CDCs having submitted NDRS report to prefecture-level CDCs in last calendar year	NA	9/14
	Proportion of CDCs having received feedback from prefecture-level CDCs in last calendar year	NA	9/9
	Proportion of CDCs having submitted NDRS report to province-level CDC in last calendar year	NA	4/14
	Proportion of CDCs having received feedback from province-level CDC in last calendar year	NA	¾
	Proportion of CDCs having submitted NDRS report to CCDC in last calendar year	26/28	NA
	Proportion of CDCs having received feedback from CCDC in last calendar year	22/26	NA

NA means not applicable.

**Table 3:** Support activities of NDRS in province-level and county-level CDCs in China

Support activities	Indicators	At province level	At county level
Training	Proportion of CDCs of which NDRS staff having been trained on communicable disease surveillance	23/28	10/14
	Proportion of CDCs of which NDRS staff having been trained in last calendar year	28/28	14/14
	Proportion of CDCs having provided training courses to other institutions in last calendar year	26/28	11/14
Supervision	Proportion of CDCs having been supervised in last calendar year	26/28	10/14
	Proportion of CDCs having made supervision visits in last calendar year	23/28	13/14
Equipment support	Proportion of CDCs having had adequate equipment for NDRS	6/28	3/14
Financial support	Proportion of CDCs having had routine budget for NDRS management	20/28	5/14
	Proportion of CDCs of which NDRS staff having been pained for overtime works	10/28	4/14

### The activities of disease-specific systems

A total of 406 province-level and 85 county-level questionnaires completed by the directors of the administrative branches of disease-specific surveillance systems were analyzed. A total of 47 diseases or syndromes were under surveillance at province level, and 20 diseases or syndromes were under surveillance at county level. Surveillance systems implemented in more than 5 CDCs at the same level are listed in table 4 and table 5.

**Table 4:** Activities of the main disease-specific surveillance systems in province-level CDCs in China

Diseases under surveillance	Indicators: proportions of CDCs having taken the activity among all surveyed CDCs at province level							
		
	Core activities				Support activities			
	
	Evaluate the data from data sources	Analyze surveillance data regularly	Submit data to CCDC regularly	Received feedbacks from CCDC in last calendar year	Made supervision visits in last calendar year	Provided training courses to other institutions in last calendar year	Have adequate equipment for surveillance	Surveillance staff have been paid for overtime works
Dengue Fever	5/5	5/5	5/5	5/5	4/5	3/5	1/5	0/5
Diarrhea caused by O157:H7	6/6	6/6	5/6	5/5	6/6	6/6	0/6	0/6
Schistosomiasis	6/6	6/6	6/6	6/6	6/6	6/6	0/6	1/6
Leptospirosis	9/9	9/9	7/9	7/7	8/9	7/9	5/9	0/9
Rabies	5/9	9/9	8/9	4/8	5/9	8/9	2/9	0/9
Typhoid	9/9	9/9	9/9	9/9	9/9	7/9	2/9	0/9
Bacillary dysentery	11/11	11/11	9/11	9/9	11/11	10/11	1/11	0/11
Malaria	12/13	13/13	13/13	13/13	13/13	12/13	2/13	1/13
Meningococcal meningitis	9/12	12/12	10/12	2/10	6/12	8/12	4/12	0/12
Encephalitis B	12/17	17/17	17/17	6/17	12/17	12/17	8/17	0/17
Brucellosis	17/17	15/17	15/17	7/15	15/17	12/17	3/17	0/17
Plague	19/19	17/19	15/19	15/15	19/19	18/19	6/19	5/19
Epidemic hemorrhagic fever	4/19	18/19	16/19	16/16	17/19	19/19	6/19	0/19
Tuberculosis	22/22	22/22	22/22	22/22	22/22	20/22	7/22	0/22
Cholera	17/22	21/22	20/22	20/20	22/22	19/22	3/22	0/22
Neonatal tetanus	18/18	17/18	15/18	15/15	14/18	13/18	6/18	0/18
AFP	25/25	25/25	25/25	25/25	25/25	24/25	7/25	2/25
AIDS	29/29	29/29	29/29	29/29	26/29	28/29	8/29	0/29
Influenza like illness	27/27	24/27	27/27	10/27	25/27	27/27	4/27	0/27
Measles	26/26	26/26	25/26	25/25	24/26	25/26	11/26	0/26

**Table 5:** Activities of the main disease-specific surveillance systems in county-level CDCs in China

Diseases under surveillance	Indicators: proportions of CDCs having taken the activity among all surveyed CDCs at county level								
		
	Core activities					Support activities			
	
	Collect samples and/or perform lab-testing	Evaluate the data from data sources	Analyze surveillance data regularly	Submit data to upper-level CDCs regularly	Received feedbacks from upper-level CDCs in last calendar year	Made supervision visits in last calendar year	Provided training courses to other institutions in last calendar year	Have adequate equipment for surveillance	Surveillance staff have been paid for overtime works
STD	1/5	3/5	5/5	5/5	2/5	5/5	2/5	2/5	0/5
Cholera	3/6	6/6	5/6	5/6	2/5	6/6	5/6	2/6	0/6
Neonatal tetanus	NA	7/7	4/7	1/7	1/1	4/7	3/7	0/7	0/7
AIDS	9/9	4/9	7/9	7/9	3/7	8/9	6/9	4/9	1/9
Measles	9/10	10/10	9/10	7/10	2/7	9/10	9/10	2/10	0/7
AFP	11/11	11/11	8/11	5/11	2/5	10/11	7/11	2/11	2/11
Tuberculosis	13/13	13/13	7/13	9/13	6/9	11/13	8/13	4/13	0/13

NA means not applicable.

### Comments on the performance of communicable disease surveillance systems from the chief directors of CDCs

29 chief directors from province-level CDCs and 14 from county-level CDCs provided their personal comments on the performance of communicable disease surveillance systems. 100% of the province-level directors and 85.7% of the county-level directors considered that NDRS had achieved its original objectives and functioned generally well. Inadequate hardware equipment, such as lack of computers or internet access, was listed as the major reason of incomplete and delayed reports by two county-level directors. Of all the disease-specific surveillance systems, AFP surveillance system received the highest recognition both at province level and county level. The chief directors from 17.2% of province-level CDCs and 35.7% of county-level CDCs considered that AFP system had achieved its objectives and functioned well. The measles surveillance system also received high recognition at province level. 13.8% of province-level directors considered that it had generally functioned well. In addition, 28.6% of county-level directors considered that TB surveillance system had functioned well.

## Discussions

### Some activities should be strengthened in NDRS

In China, notifiable disease reporting is mandatorily required by the Law of the People's Republic of China on Prevention and Treatment of Infectious Diseases. An internet-based real-time information platform has been established to facilitate case reporting of notifiable disease. To monitor the performance of NDRS, CCDC has developed a series of indicators including timeliness, proportions of duplicated and missing reports. Evaluations showed that the performance had been dramatically improved since 2004 [23]. In light of the results of this survey, almost all the CDCs have had the main activities of NDRS like confirmation, analysis, reporting, training and supervision, which was consistent with the conclusion of annual quality analysis report of NDRS published by the Information Center of CCDC[8]. But some activities, including feedback, equipment and overtime pay providing need to be strengthened.

Feedback is one of the most important elements of surveillance cycle [24]. Findings in this survey indicate that the information loop of NDRS was incomplete due to the absence of feedback, especially the feedback from decision makers (or higher level of the surveillance system) to data providers (or lower level of the surveillance system). But the situations varied among different levels and institutions. In general, the information loop inside the CDC system was complete. The proportions of feedback from higher level CDCs to the lower level CDCs were all greater than 60%, but the proportions of feedback from health administrative departments to CDCs were lower than 30% at all levels. In China, the health administrative departments have significant influence on the activities of CDCs. The performance and the quality of communicable disease surveillance activities of CDCs might be compromised by the lack of clear and incentive feedback from the health administrative departments. Unfortunately, the existing regulations and guidelines do not oblige health administrative departments to give CDCs essential feedback.

Less than 30% of the CDCs, at either province level or county level, thought that their equipment had met the demands of NDRS. Especially the computers equipped at the beginning of the internet-based real-time report system have been worn-down after years of heavy work [25]. However, most of the surveyed CDCs reported that they could not afford equipment replacement. Less than 40% of the province-level CDCs and 30% of the county-level CDCs reported they had paid the overtime work of NDRS, and this could be an important negative factor affecting the enthusiasm of working staff.

### Performance varied among disease-specific surveillance systems

The results showed that the activities among different disease-specific surveillance systems varied widely, which was in accordance with the comments on the performance of communicable disease surveillance systems from the directors of CDCs. AFP, measles and TB surveillance systems got relatively high recognition. A clear target of prevention and control, especially when the target was announced by government or international organizations, as well as the long-term assistance from large-scale intervention programs both were positive factors for disease-specific surveillance systems, which was highly consistent with the situations in other countries [16-19].

### More comprehensive and in-depth descriptions and evaluations of communicable disease surveillance systems in China are needed

Several guidelines or protocols about how to evaluate the public health surveillance or the communicable disease surveillance systems had been developed by US CDC and WHO since 1980 s. The elements of evaluation were firstly described by US CDC in the guideline published in 1988, including the public health importance of the disease or health conditions under surveillance, the framework of the system, the attributes of the surveillance system (simplicity, flexibility, acceptability, sensitivity, positive predictive value, representativeness, and timeliness), and the usefulness and cost [26,27]. In 2001, WHO released a protocol for the assessment of national communicable disease surveillance and response system and updated it in 2006[21,22]. Besides the elements mentioned in the US CDC guideline, the WHO protocol also emphasized the evaluation should also pay attention to the functions of surveillance systems and seek for the integration opportunity [15,21,22].

The idea of surveillance system evaluation was introduced into China in the early of 1990 s soon after the first US CDC guideline released [6,7], and this guideline had a big impact on the evaluation of surveillance system in China. Most of the evaluations were focused on attributes of completeness and timeliness. Unfortunately most of the efforts had been concentrated on a small number of systems such as NDRS, influenza system, and AFP system, while other systems were seldom evaluated. Such results helped us to promote the evaluated systems, but if we want to draw up a plan to enhance the overall regional or national communicable disease surveillance systems, the evaluation of limited systems and limited attributes would not provide strong and comprehensive evidence for decision making. In particular, when integration is planned, which has been appealed repeatedly, the evaluation result of the whole system is essential. Evaluating how the systems function, identifying where the synergy is possible, finding which can be the driving force, and determining whether national surveillance systems can be integrated is the spirit of WHO infectious disease integrated strategy which had been used in Africa [28]. This strategy has led to a great success in helping the member countries in AFRO to enhance their national communicable disease surveillance and response systems [29]. Given that China has much more complicated demographic, economical and social conditions than African countries, the more the in-depth comprehensive evaluations, the better the evidence would be to help the improvement of the national communicable disease surveillance and response system. This study is just the first stage in a complete description and evaluation of China's communicable disease surveillance systems.

## Conclusions

China has already established a national communicable disease surveillance framework which was comprised of NDRS and disease-specific surveillance systems. As the core system for the communicable disease surveillance in China, NDRS has generally functioned well. But the performance varies among different disease-specific surveillance systems. Some activities need to be strengthened and more in-depth and comprehensive descriptions and evaluations are needed to improve the communicable disease surveillance systems in China.

## Powers and Limitations

This study is the first systematic description of communicable disease surveillance systems at operating level in China. It is also China's first nationwide application of the conceptual framework of surveillance proposed by WHO and US CDC, which divided the activities of surveillance into two parts: core and support activities. The authors developed definition for core and support activities of communicable disease surveillance based on China's national condition and modified the assessment protocol published by WHO. The methodology and the results lay a basis for future investigations in this field.

Although the questionnaire used in this study was designed based on the well recognized conceptual framework of surveillance proposed by WHO and US CDC, the authors did many modifications to make it more suitable for the research purpose, that is, to learn how the existing surveillance systems worked at the province-level and county-level CDCs in China. That's the reason why almost all the items about hospitals, health administrative departments and CCDC were excluded. Because the subsequent actions following surveillance data like outbreak investigation, response to epidemic and epidemic preparedness were not the main concern either, items about those parts were minimized. So it may be inappropriate to direct compare the findings described in this paper with the results of communicable disease surveillance system evaluations from other countries due to the different data sources and study interests.

There are several limitations in this study. Firstly, respondents were asked to describe the surveillance works of past year in details by themselves, so the recall bias is probably unavoidable. Secondly, being restricted by insufficient database in the sampling of county-level CDCs, shortage of support and difficulties in implementation, the authors only investigated 14 county-level CDCs as a typical sample. Although the results from surveyed county-level CDCs can reflect most others' situation to some extent, the selection bias still can not be ignored.

Due to the lack of proper references, it is difficult to estimate either the size or the direction of the biases. Future systematic descriptions or evaluations of communicable disease surveillance systems in China could provide more information to complement the findings. The findings described in this paper may not be applicable to all county-level CDCs in China and the results must be used cautiously. Given that all the province-level CDCs were investigated and the response rate (91.9%) and invalid proportion (6.9%) were acceptable, the results from province-level CDCs could represent the true situation for this level.

## Competing interests

The authors declare that they have no competing interests.

## Authors' contributions

WYX and LML initiated this study. WYX carried out the design of the study, the mailed investigation, data entry, data analysis and paper writing. JL participated in the design of the study, data analysis and paper writing. LML participated in the design of the study, carried out the paper reviewing. All authors read and approved the final manuscript.

## Pre-publication history

The pre-publication history for this paper can be accessed here:

http://www.biomedcentral.com/1471-2458/10/704/prepub

## Supplementary Material

Additional file 1 **Questionnaire for director of province-level CDC**. The questionnaire for the chief directors of province-level CDCs. It comprised 13 questions including the list of priority communicable disease, the goal of communicable disease prevent and control, the current situation of NDRS and disease-specific surveillance systems and the comments. Click here for file

Additional file 2 **Questionnaire for NDRS at province-level**. The questionnaire for the directors of the NDRS administrative branches at province-level CDCs. It comprised 24 questions. All the related activities presented in table 1 were included. Click here for file

Additional file 3 **Questionnaire for disease-specific surveillance systems at province-level CDCs**. The questionnaire for the directors of the administrative branches of disease-specific surveillance systems at province-level CDCs. It comprised 25 questions. All the related activities presented in table 1 were included. Click here for file

Additional file 4 **Questionnaire for director of county-level CDC**. The questionnaire for the chief directors of county-level CDCs. It comprised 13 questions including the list of priority communicable disease, the goal of communicable disease prevent and control, the current situation of NDRS and disease-specific surveillance systems and the comments. Click here for file

Additional file 5 **Questionnaire for NDRS at county-level CDCs**. The questionnaire for the directors of the NDRS administrative branches at county-level CDCs. It comprised 23 questions. All the related activities presented in table 1 were included. Click here for file

Additional file 6 **Questionnaire for disease-specific surveillance systems at county-level CDCs**. The questionnaire for the directors of the administrative branches of disease-specific surveillance systems at county-level CDCs. It comprised 25 questions. All the related activities presented in table 1 were included. Click here for file
