# Supplementary material for: A survey of core and support activities of communicable disease surveillance systems at operating-level CDCs in China
Source: BMC Public Health. 2010 Nov 17;10:704. doi: 10.1186/1471-2458-10-704 (PMC2996372; doi:10.1186/1471-2458-10-704)
Supplement: Additional file 3 — Questionnaire for disease-specific surveillance systems at province-level CDCs. The questionnaire for the directors of the administrative branches of disease-specific surveillance systems at province-level CDCs. It comprised 25 questions. All the related activities presented in table 1 were included. [file 1471-2458-10-704-S3.DOC]

**Questionnaire Number:**

**Questionnaire for disease-specific surveillance systems at province-level CDC**

**Date: (year/month/day)**

**Province:**

**Branch:**

**Respondent name: Tel:**

**Background**

This questionnaire is developed by the Department of Epidemiology and Biostatistics, School of Public Health, Peking University Health Science Center. The purpose of this investigation is to describe the activities of communicable disease surveillance systems in China. All the information collected is only for policy analysis and will not be used for any commercial purposes. All the personal- and organizational-specific information will not be released in any reports unless approved.

Please fill out the questionnaire in sequence of the question numbers. There are three types of questions: 1) fill-in-the-blank. Please complete the text or fill the empty table following the instructions; 2) choice question. All the choice questions are single-choice unless special instructions are given. Please answer these questions by checking off the choice that best match your agency’s situation; 3) essay and opening question. Please use the margin of both pages to elaborate on your answers.

All the information you provided are very important to the investigation. Thanks for your participation and help!

**Part I General information**

**GQ1. Describe the disease-specific surveillance systems managed by your branch. Use the margin when needed.**

**System 1: ________________________________________________________________________**

**System 2: ________________________________________________________________________**

**System 3: ________________________________________________________________________**

**System 4: ________________________________________________________________________**

**System 5: ________________________________________________________________________**

**GQ2. Describe the NUMBER of employees working for disease-specific surveillance systems in your branch.**

*Duty description*: ① data collection and analysis ② network management and maintenance ③ others.

*Education level*: ① senior high school and below ② technical secondary school

③ junior college or bachelor degree ④ master degree and above

*Specialty of highest degree*: ① public health ② clinical medicine ③ laboratory medicine ④ nursing ⑤ IT

⑥ others ⑦ none of above

*Years of service*: only the years working for NDRS are accounted. 1/2 can be used to describe the years.

|  | Duty description | | | Education level | | | | Specialty of highest degree | | | | | | | Years of service |
| --- | --- | --- | --- | --- | --- | --- | --- | --- | --- | --- | --- | --- | --- | --- | --- |
|  | ① | ② | ③ | ① | ② | ③ | ④ | ① | ② | ③ | ④ | ⑤ | ⑥ | ⑦ |  |
| Full-time |  |  |  |  |  |  |  |  |  |  |  |  |  |  |  |
| Part-time |  |  |  |  |  |  |  |  |  |  |  |  |  |  |  |

**GQ3. Is there a working system of disease-specific surveillance system management in your branch?**

| ① Yes, it has been launched for ______ year(s) | ② No | ③ I don’t know |
| --- | --- | --- |

**Part II Disease-specific surveillance system**

**Disease under surveillance: ________________________________________________________**

| **Q1. Are there any intervention programs on this disease in your province? If yes, describe the program(s):** |
| --- |

| Supported by provincial fund | Supported by national fund | Supported by international fund |
| --- | --- | --- |
|  |  |  |
|  |  |  |
|  |  |  |
|  |  |  |

| **Q2. Are there any outbreaks of this disease in your province during last calendar year?** |
| --- |

| ① Yes, go to Q2.1 | ② No, go to Q3 | ③ I don’t know, go to Q3 |
| --- | --- | --- |

**Q2.1** There were________( number of outbreaks) outbreaks of this disease within last calendar year. Your branch responded to _______ (number of outbreaks) of them, and drafted _______ (number of reports) outbreak investigation reports.

| **Q3. Describe the coverage of this disease-specific surveillance system in your province.** |
| --- |

| ① The whole province, go to Q4 | ② Only surveillance sites, go to Q3.1 |
| --- | --- |
| ③ Other, please specify:____________________________________________________________ | |

**Q3.1** There are ______ (number of sites) surveillance sites in the province. ______(number of sites) of these sites are supported by national fund, ______ (number of sites) are supported by provincial fund.

| **Q4. What is the leading objective of this surveillance system?** |
| --- |

① Early detection of outbreaks so as to make responses in time.

② Systematical description of epidemiology of disease and the changes in the related factors so as to provide evidence for long-term policy making

③ Evaluating intervention program(s). Specify the program(s):

________________________________________________________________________________

④ Other, please specify: ____________________________________________________________

| **Q5. Describe the working pattern of this disease-specific surveillance system.** | | |
| --- | --- | --- |
| ① Positive surveillance | ② Passive surveillance | ③ Combination of positive and passive |

| **Q6. Describe the data source of this surveillance system（chose all apply）** | | |
| --- | --- | --- |
| ① General hospitals | ② Community health centers | ③ Special hospitals |
| ④ Other, please specify: ____________________________________________________________ | | |

| **Q7. Describe the surveillance contents.**  If it is unseasonal work, check√ the suitable one; if it is seasonal work, specify the duration, e.g.: 10.1－3.31 |
| --- |

|  | Cases surveillance | Syndromic surveillance | Serologic surveillance | Behavior surveillance | Pathogen surveillance | Host surveillance | Vector surveillance |
| --- | --- | --- | --- | --- | --- | --- | --- |
| Unseasonal |  |  |  |  |  |  |  |
| Seasonal |  |  |  |  |  |  |  |
|  | | | | | | | |

| **Q8. Are there any national guidelines for this surveillance system?** |
| --- |

| ① Yes, go to Q8.1 | ② No, go to Q9 | ③ I don’t know, go to Q9 |
| --- | --- | --- |

**Q8.1 Describe these guidelines. Specify the titles and the organizations issuing the guideline.**

**_______________________________________________________________________________**

**_______________________________________________________________________________**

| **Q9. Are there any unified format data collection forms used in this surveillance system？** |
| --- |

| ① Yes, go to Q9.1 | ② No, go to Q10 | ③ I don’t know, go to Q10 |
| --- | --- | --- |

**Q9.1 Describe these forms. Specify the titles and the organizations developing these forms.**

**_______________________________________________________________________________**

**_______________________________________________________________________________**

| **Q10. Have the surveillance staffs been requested to collect biological samples, e.g. blood, urine, CSF?** |
| --- |

| ① Yes, go to Q10.1 | ② No, go to Q11 | ③ I don’t know, go to Q11 |
| --- | --- | --- |

**Q10.1 Describe these samples.**

**________________________________________________________________________________**

| **Q11. Describe the time limit of the surveillance data reporting. Check  the suitable one.** |
| --- |

| 1. From lower-level CDCs or hospitals to your branch | | | | | | | |
| --- | --- | --- | --- | --- | --- | --- | --- |
|  | Real-time/daily | Weekly | Every 10 day | Monthly | Quarterly | Annually | Other, please specify: |
| Results of lab test |  |  |  |  |  |  |  |
| Other data |  |  |  |  |  |  |  |
| 2. From your branch to China CDC | | | | | | | |
|  | Real-time/daily | Weekly | Every 10 day | Monthly | Quarterly | Annually | Other, please specify: |
| Results of lab test |  |  |  |  |  |  |  |
| Other data |  |  |  |  |  |  |  |

| **Q12. Describe the mechanism of the surveillance data reporting. Check  the suitable one. If the internet-based reporting is used, specify the name of the reporting system.** |
| --- |

| 1. From lower-level CDC or hospitals to your branch | | | | | | |
| --- | --- | --- | --- | --- | --- | --- |
|  | Internet-based reporting | By post | Email | Telephone | Fax | Other, please specify: |
| Results of lab test |  |  |  |  |  |  |
| Other data |  |  |  |  |  |  |
| 2. From your branch to China CDC | | | | | | |
|  | Internet-based reporting | By post | Email | Telephone | Fax | Other, please specify: |
| Results of lab test |  |  |  |  |  |  |
| Other data |  |  |  |  |  |  |

| **Q13. Does your branch take the responsibility of surveillance data analysis?** |
| --- |

| ① Yes, go to Q13.1-13.2 | ② No, go to Q14 | ③ I don’t know, go to Q14 |
| --- | --- | --- |

**Q13.1** **Describe the source of denominators:**

**________________________________________________________________________________**

**Q13.2 Describe the analysis. Check  the suitable one.**

| Periodicity of analysis | Data analysis | | | | | | |
| --- | --- | --- | --- | --- | --- | --- | --- |
|  | Completeness of the data | Timeliness of the data | Describe data by person\place\time | Trend analysis | Predictive analysis | Risk factors analysis | Other, please specify: |
| Non-periodically |  |  |  |  |  |  |  |
| Daily |  |  |  |  |  |  |  |
| Weekly |  |  |  |  |  |  |  |
| Every 10 day |  |  |  |  |  |  |  |
| Monthly |  |  |  |  |  |  |  |
| Quarterly |  |  |  |  |  |  |  |
| Every half year |  |  |  |  |  |  |  |
| Annually |  |  |  |  |  |  |  |

| **Q14. Has your branch ever been requested to submit written report of surveillance data?** | | |
| --- | --- | --- |
| ① Yes, go Q14.1-14.2 | ② No, go Q15 | ③ I don’t know, go Q15 |

**Q14.1 Describe the organizations to which reports were submitted. Check  the suitable one**

| Organization | Report submitted | | Periodicity of report submission | | | | | The number of the reports submitted in last calendar year |
| --- | --- | --- | --- | --- | --- | --- | --- | --- |
|  | Yes | No | Weekly | Monthly | Quarterly | Annually | Other, please specify: |  |
| Province health administration department |  |  |  |  |  |  |  |  |
| China CDC |  |  |  |  |  |  |  |  |
| MOH |  |  |  |  |  |  |  |  |
| Other, please specify: |  |  |  |  |  |  |  |  |

**Q14.2 Describe the feedbacks received within last calendar year. Check  the suitable one.**

| Organization | Feedback | | Forms of feedback | | | | The number of feedbacks received in last calendar year |
| --- | --- | --- | --- | --- | --- | --- | --- |
|  | Yes | No | Phone call | Official report | Bulletin | Other, please specify: |  |
| Province health administration department |  |  |  |  |  |  |  |
| China CDC |  |  |  |  |  |  |  |
| MOH |  |  |  |  |  |  |  |
| Other, please specify: |  |  |  |  |  |  |  |

| **Q15. Have your branch ever been supervised during last calendar year for disease-specific surveillance work?** |
| --- |

| ① Yes, go to Q15.1 | ② No, go to Q 16 | ③ I don’t know, go to Q16 |
| --- | --- | --- |

**Q15.1 Describe the supervisions within last calendar year. Specify the supervisors.**

________________________________________________________________________________

________________________________________________________________________________

________________________________________________________________________________

________________________________________________________________________________

| **Q16. Did your branch made supervisory visits during last calendar year?** |
| --- |

| ① Yes, go to Q16.1 | ② No, go to Q17 | ③ I don’t know, go to Q17 |
| --- | --- | --- |

**Q16.1 Describe the supervisory visits made by your branch within last calendar year. Specify the types and the numbers of supervised institutions.**

________________________________________________________________________________

________________________________________________________________________________

________________________________________________________________________________

________________________________________________________________________________

| **Q17.Have the working staffs in your branch ever been trained on disease-specific surveillance?** |
| --- |

| ① Yes, specify how long:_____________ | ② No, go to Q18 | ③ I don’t know, go to Q18 |
| --- | --- | --- |

| **Q18. Describe the training courses received by the working staffs in your branch within last calendar year.** |
| --- |

| Training | Total trained person-times | Contents of the training(s) |
| --- | --- | --- |
| Province-level |  |  |
| National |  |  |
| International |  |  |

| **Q19. Have your branch provided trainings about this disease-specific surveillance in last calendar year?** |
| --- |

| ① Yes, go to Q19.1 | ② No, go to Q20 | ③ I don’t know, go to Q20 |
| --- | --- | --- |

**Q19.1 Describe the trainings courses provided by your branch within last calendar year. Specify the total number of trained persons and the training contents.**

________________________________________________________________________________

________________________________________________________________________________

________________________________________________________________________________

________________________________________________________________________________

| **Q20. List the equipments used for this disease-specific surveillance in your branch** |
| --- |

| Does your branch have access to internet? ① Yes, it is stable ② Yes, but it is unstable ③ No | | | | | | |
| --- | --- | --- | --- | --- | --- | --- |
| Equipment | Total  number | Are they working well? | | | Do they need updating? | |
|  |  | Fully or almost fully | Partially | Not at all | Yes | No |
| Computer |  |  |  |  |  |  |
| Laptop |  |  |  |  |  |  |
| Fixed phone |  |  |  |  |  |  |
| Fax |  |  |  |  |  |  |
| Printer |  |  |  |  |  |  |

**Q20.1 Do you think the existing equipment can satisfy the demand of this disease-specific surveillance or not?**

| ① Yes | ② No | ③ I don’t know |
| --- | --- | --- |

| **Q21. Do you know your branch’s funding source of this disease-specific surveillance?** | |
| --- | --- |
| ① Yes, go to 21.1 | ② No, go to Q22 |

**Q21.1** **Describe the funding sources of last calendar year.**

Total fund is ________________________ Yuan RMB

Source 1 _________________________ (title) afforded ______________________ Yuan RMB

Source 2 _________________________ (title) afforded ______________________ Yuan RMB

Source 3 _________________________ (title) afforded ______________________ Yuan RMB

| **Q22. Do you know your branch’s expenditure on this disease-specific surveillance?** | |
| --- | --- |
| ① Yes, go to Q22.1-22.2 | ② No, go to Q23 |

**Q22.1 Specify the percentages of each items of expenditure on this disease-specific surveillance within last calendar year (%).**

| Communication | Equipment | Travel | Training | | Office supplies | Allowance | Other |
| --- | --- | --- | --- | --- | --- | --- | --- |
|  |  |  |  |  | |  |  |

**Q22.2 Do you think the existing financial support can satisfy the demand of this disease-specific surveillance or not?**

| ① Yes | ② No | ③ I don’t know |
| --- | --- | --- |

| **Q23. Do the working staffs in your branch have overtime pay or paid leave for working for this disease-specific surveillance on weekends or holidays?** |
| --- |

| ① Yes, describe it:_________________________________________________________________ | |
| --- | --- |
| ② No | ③ I don’t know |

| **Q24. In your opinion, what kind of supports are needed for the disease-specific surveillance system improvement in your province?（chose all apply）** | | |
| --- | --- | --- |
| ① Policy support | ② Financial and equipment support | ③ Integration support |
| ④ Staff training | ⑤ Technique support |  |

⑥ Other, please specify: ____________________________________________________________

*Please sort the chosen supports in order of importance：___________________________________*

| **Q25. What is your suggestion to improve the performance of this disease-specific surveillance in your province？** |
| --- |

________________________________________________________________________________________________________________________________________________________________________________________________________________________________________________

________________________________________________________________________________

**Thank you for your time**

**Please send the finished questionnaire back to...... before../../..**
