# Supplementary material for: A survey of core and support activities of communicable disease surveillance systems at operating-level CDCs in China
Source: BMC Public Health. 2010 Nov 17;10:704. doi: 10.1186/1471-2458-10-704 (PMC2996372; doi:10.1186/1471-2458-10-704)
Supplement: Additional file 4 — Questionnaire for director of county-level CDC. The questionnaire for the chief directors of county-level CDCs. It comprised 13 questions including the list of priority communicable disease, the goal of communicable disease prevent and control, the current situation of NDRS and disease-specific surveillance systems and the comments. [file 1471-2458-10-704-S4.DOC]

**Questionnaire Number:**

**Questionnaire for director of county-level CDC**

**Date: (year/month/day)**

**Province: City/prefecture: County:**

**Respondent name: Tel:**

**Background**

This questionnaire is developed by the Department of Epidemiology and Biostatistics, School of Public Health, Peking University Health Science Center. The purpose of this investigation is to describe the activities of communicable disease surveillance systems in China. All the information collected is only for policy analysis and will not be used for any commercial purposes. All the personal- and organizational-specific information will not be released in any reports unless approved.

Please fill out the questionnaire in sequence of the question numbers. There are three types of questions: 1) fill-in-the-blank. Please complete the text or fill the empty table following the instructions; 2) choice question. All the choice questions are single-choice unless special instructions are given. Please answer these questions by checking off the choice that best match your agency’s situation; 3) essay and opening question. Please use the margin of both pages to elaborate on your answers.

All the information you provided are very important to the investigation. Thanks for your participation and help!

| **Q1. Describe the top 3 communicable diseases reported in your county in last calendar year** | | | |
| --- | --- | --- | --- |
|  | By incidence rate | By fatality rate | By the frequency of outbreaks |
| 1 |  |  |  |
| 2 |  |  |  |
| 3 |  |  |  |

**Q1.1 what is the data basis of your Q1 answer?**

| ① Governmental communicable disease bulletin | ② Surveillance report of CDC |
| --- | --- |
| ③ Expert advice | ④ Your personal opinion |

⑤ Other, please specify: ____________________________________________________________

| **Q2. Are there any diseases announced to be Class B or C notifiable diseases in your province besides those included in the Law of the People's Republic of China on Prevention and Treatment of Infectious Diseases?** | | |
| --- | --- | --- |
| ① Yes, go to Q2.1 | ② No, go to Q3 | ③ I don’t know, go to Q3 |

**Q2.1** Specify these diseases.

| Included in Class B |  |  |  |  |  |  |
| --- | --- | --- | --- | --- | --- | --- |
| Included in Class C |  |  |  |  |  |  |

| **Q3. Do you know the local goals of communicable disease prevention and control?** | |
| --- | --- |
| ① Yes, go to Q3.1-Q3.3 | ② There are no such goals, go to Q4 |
| ③ I don’t know, go to Q4 |  |

**Q3.1 Specify the diseases according to the goals.**

| Goal | Diseases to be controlled |
| --- | --- |
| Elimination |  |
| Control | High incidence/fatality rate: |
|  | Epidemic-prone: |
| Other |  |

**Q 3.2 Specify the organizations which set the goals.**

________________________________________________________________________________

**Q3.3 As you know, which is the immediate concern to set the goals?**

① Goals and work plans issued by WHO

② Goals and work plans issued by MOH

③ Goals and work plans issued by the province-level health administration department

④ Goals and work plans issued by Local CDC

⑤ Expert advices

⑥ Other, please specify: ____________________________________________________________

| **Q4. Which branch is in charge of NDRS system management in county-level CDC?** |
| --- |

________________________________________________________________________________

| **5. Are there any disease-specific control programs in county-level CDC?** | | |
| --- | --- | --- |
| ① Yes, go to Q5.1 | ② No, go to Q6 | ③ I don’t know, go to Q6 |

**Q5.1 Describe these programs.**

|  | Program name | Management dep. |
| --- | --- | --- |
| Supported by county fund |  |  |
|  |  |
|  |  |
| Supported by prefectural fund |  |  |
|  |  |
|  |  |
| Supported by provincial fund |  |  |
|  |  |
|  |  |
| Supported by national fund |  |  |
|  |  |
|  |  |
|  |  |
| Supported by international fund |  |  |
|  |  |
|  |  |
|  |  |

| **Q6. Has the internet-based real-time reporting mechanism been used in all the communicable disease surveillance systems in your county?** | | |
| --- | --- | --- |
| ① Yes, go to Q7 | ② No, go to Q6.1 | ③I don’t know, go to Q7 |

**Q6.1 Why this reporting** mechanism has not been used yet? （chose all the apply）

| ① Lack of policy support | ② Lack of fund and equipment | ③ Lack of recognition |
| --- | --- | --- |
| ④ Lack of staff | ⑤ Lack of collaboration | |

⑥ Other, please specify: ____________________________________________________________

| **Q7. There were_______ (number of reports) outbreaks detected by county-level CDC in last calendar year. County-level CDC responded to _______ (number of outbreaks) of them. Mean time interval between outbreak detection and response was _______ day(s).** |
| --- |

| **Q8. Does county-level CDC have routine budget for NDRS management?** | | |
| --- | --- | --- |
| ① Yes | ② No | ③ I don’t know |

| **Q9. Does county-level CDC have routine budgets for disease-specific surveillance systems?** | | | |
| --- | --- | --- | --- |
| ① Yes | ② Partially | ③ No | ④ I don’t know |

| **Q10. In your opinion, _______________ (system name) is the most well-functioned disease-specific surveillance system. Describe the reason why it is functioning well.（chose all apply）** | |
| --- | --- |
| ① The disease under surveillance has high priority | ② Strong policy support |
| ③ Sufficient fund | ④ High system acceptability |
| ⑤ Motivated and engaged workforce |  |

⑥ Other, please specify: ____________________________________________________________

*Please sort the chosen reasons in order of importance：____________________________________*

| **Q11. As your know, has the communicable disease surveillance data ever been used in any area?（chose all apply）** |
| --- |

① Local policy making

② National policy making

③ Advocacy

④ Evaluation of prevention and control programs

⑤ Request for funds

⑥ Other, please specify: ____________________________________________________________

| **Q12. In your opinion, what are the problems in the surveillance of communicable disease in your county?（chose all apply）** | |
| --- | --- |
| ① Lack of policy support | ② Lack of fund and equipment |
| ③ Lack of integration | ④ Lack of training staff |
| ⑤ Lack of technique support |  |

⑥ Other, please specify: ____________________________________________________________

*Please sort the chosen problems in order of importance：___________________________________*

| **Q13. What is your suggestion to improve the performance of communicable disease surveillance in your county?** |
| --- |

________________________________________________________________________________________________________________________________________________________________________________________________________________________________________________

________________________________________________________________________________

**Thank you for your time**

**Please send the finished questionnaire back to...... before../../..**
