# Supplementary material for: A survey of core and support activities of communicable disease surveillance systems at operating-level CDCs in China
Source: BMC Public Health. 2010 Nov 17;10:704. doi: 10.1186/1471-2458-10-704 (PMC2996372; doi:10.1186/1471-2458-10-704)
Supplement: Additional file 5 — Questionnaire for NDRS at county-level CDCs. The questionnaire for the directors of the NDRS administrative branches at county-level CDCs. It comprised 23 questions. All the related activities presented in table 1 were included. [file 1471-2458-10-704-S5.DOC]

**Questionnaire Number:**

**Questionnaire for NDRS at county-level CDC**

**Date: (year/month/day)**

**Province: City/prefecture: County:**

**Branch:**

**Respondent name: Tel:**

**Background**

This questionnaire is developed by the Department of Epidemiology and Biostatistics, School of Public Health, Peking University Health Science Center. The purpose of this investigation is to describe the activities of communicable disease surveillance systems in China. All the information collected is only for policy analysis and will not be used for any commercial purposes. All the personal- and organizational-specific information will not be released in any reports unless approved.

Please fill out the questionnaire in sequence of the question numbers. There are three types of questions: 1) fill-in-the-blank. Please complete the text or fill the empty table following the instructions; 2) choice question. All the choice questions are single-choice unless special instructions are given. Please answer these questions by checking off the choice that best match your agency’s situation; 3) essay and opening question. Please use the margin of both pages to elaborate on your answers.

All the information you provided are very important to the investigation. Thanks for your participation and help!

| **Q1. Describe the NUMBER of employees working for NDRS in your branch.** |
| --- |

*Duty description*: ① data collection and analysis ② network management and maintenance ③ others.

*Education level*: ① senior high school and below ② technical secondary school

③ junior college or bachelor degree ④ master degree and above

*Specialty of highest degree*: ① public health ② clinical medicine ③ laboratory medicine ④ nursing ⑤ IT

⑥ others ⑦ none of above

*Years of service*: only the years working for NDRS are accounted. 1/2 can be used to describe the years.

|  | Duty description | | | Education level | | | | Specialty of highest degree | | | | | | | Years of service |
| --- | --- | --- | --- | --- | --- | --- | --- | --- | --- | --- | --- | --- | --- | --- | --- |
|  | ① | ② | ③ | ① | ② | ③ | ④ | ① | ② | ③ | ④ | ⑤ | ⑥ | ⑦ |  |
| Full-time |  |  |  |  |  |  |  |  |  |  |  |  |  |  |  |
| Part-time |  |  |  |  |  |  |  |  |  |  |  |  |  |  |  |

| **Q2. Do you have any guidelines for NDRS management besides the Law of the People's Republic of China on Prevention and Treatment of Infectious Diseases?**  **If yes, describe these documents and go to Q2.1. If no, go to Q3.** | | |
| --- | --- | --- |
| Title | Publisher | Issue time(M/Y) |
|  |  |  |
|  |  |  |
|  |  |  |
|  |  |  |
|  |  |  |
|  |  |  |
|  |  |  |
|  |  |  |
|  |  |  |
|  |  |  |

**Q2.1 What is the deficiency of these documents when applied to use? (chose all the apply)**

| ① There are some duplications among different guidelines | ② Lack of practicability |
| --- | --- |
| ③ There are some conflicts among different guidelines | ④ Some contents are ambiguously phrased |
| ⑤ Other, please specify: __________________________________________________________________ | |

*Please sort the chosen deficiencies in order of importance：_________________________________*

| **Q3. Is there a working system of NDRS management in your branch?** | | |
| --- | --- | --- |
| ① Yes, it has been launched for ______ year(s) | ② No, go to Q4 | ③ I don’t know, go to Q4 |

| **Q4. Has computerized surveillance network been used in NDRS in your county?** | | |
| --- | --- | --- |
| ① Yes, go to Q4.1-4.2 | ② No, go to Q4.3 | ③ I don’t know, go to Q5 |

**Q4.1 Describe the network(s) (chose all the apply ):**

① The internet-based real-time reporting system established by China CDC

② The reporting system established by province-level CDC. Specify the name of the system:

________________________________________________________________________________

③ The reporting system established by province-level CDC. Specify the name of the system:

________________________________________________________________________________

④ Other, please specify: ____________________________________________________________

| **4.2 Fill the table below with the latest data.** | | | | | | |
| --- | --- | --- | --- | --- | --- | --- |
|  | Number of the institutions | | Number of the institutions with the ability of NDRS internet-based real-time reporting | Number of the institutions without the ability of NDRS internet-based real-time reporting | | Number of the institutions has made NDRS internet-based real-time reporting in last 3 months |
| Hospitals |  | |  |  | |  |
| Health centers |  | |  |  | |  |
| Other medical facilities |  | |  |  | |  |
| **Q4.3 Describe the data reporting mechanism of NDRS in your county:**  _________________________________________________________________________________ | | | | | | |
| **Q5. Are there any other reporting cards used in NDRS in your county besides the national notifiable disease reporting card?** | | | | | | |
| ① Yes, go to Q5.1 | | ② No, go to Q6 | | | ③ I don’t know, go to Q6 | |

**Q5.1 Describe these cards.**

| Title | Publisher | Issue time (M/Y) |
| --- | --- | --- |
|  |  |  |
|  |  |  |
|  |  |  |

| **Q6. How did your branch notice the latest outbreak of notifiable disease in your county?** | |
| --- | --- |
| ① From the report of local CDC | ② Informed by local health bureau |
| ③ From routine data analysis of your branch | ④ Informed by other higher authorities |
| ⑤ From the public media | ⑥ From public hotline |

⑦ Other, please specify: ____________________________________________________________

| **Q7. Does your branch take the responsibility of NDRS data analysis?** | |
| --- | --- |
| ① Yes, go to Q7.1-7.2 | ② No, ______________(branch name) take the responsibility, go to Q 8 |

**Q7.1 Describe the source of denominator:**

**________________________________________________________________________________**

**Q7.2 Describe the content of analysis. Check  the suitable one.**

| Periodicity of analysis | | Content of analysis | | | | | | |
| --- | --- | --- | --- | --- | --- | --- | --- | --- |
|  |  | Data summarizing | Describe data by person\place\time | Cluster analysis | Trend analysis | Predictive analysis | Risk factor analysis | Reporting quality analysis |
| Non-periodically | |  |  |  |  |  |  |  |
| periodically | Daily |  |  |  |  |  |  |  |
|  | Weekly |  |  |  |  |  |  |  |
|  | Every 10 day |  |  |  |  |  |  |  |
|  | Monthly |  |  |  |  |  |  |  |
|  | Quarterly |  |  |  |  |  |  |  |
|  | Every half year |  |  |  |  |  |  |  |
|  | Annually |  |  |  |  |  |  |  |

| **Q8. Does your branch take the responsibility of NDRS reporting quality evaluation?** | | |
| --- | --- | --- |
| ① Yes, go to Q8.1 | ② No, go to Q8.2 | ③ I don’t know, go to Q9 |

**Q8.1 Describe the evaluations. Check  the suitable** one.

| Periodicity of evaluation | | Content of evaluation | | | | | | |
| --- | --- | --- | --- | --- | --- | --- | --- | --- |
|  |  | Number of unverified cards | Number of duplicated cards | Timeliness of report | Timeliness of verifying | Completeness of report | Composite index | Other, please specify: |
| Non-periodically | |  |  |  |  |  |  |  |
| Periodically | Daily |  |  |  |  |  |  |  |
|  | Weekly |  |  |  |  |  |  |  |
|  | Every 10 day |  |  |  |  |  |  |  |
|  | Monthly |  |  |  |  |  |  |  |
|  | Quarterly |  |  |  |  |  |  |  |
|  | Every half year |  |  |  |  |  |  |  |
|  | Annually |  |  |  |  |  |  |  |

**Q8.2 Describe the most usual reason for not evaluating the reporting quality of NDRS.**

① It is other branch’s responsibility to do the evaluation. The branch is ______________________

| ② It is not required | ③ It is not necessary | ④ Lack of working staff |
| --- | --- | --- |

⑤ Other, please specify :____________________________________________________________

| **Q9. The mean completeness of NDRS reporting in your county of last calendar year is___________________. The mean timeliness is ___________________.** |
| --- |

| **Q10. Have there been any investigations of NDRS missing report in your county?** | | |
| --- | --- | --- |
| ① Yes, **go to Q10.1-10.2** | ② No, **go to Q10.2** | ③ I don’t know, go to Q11 |

**Q10.1 Describe the investigations within last three years.**

| Year | In hospitals | | In communities | |
| --- | --- | --- | --- | --- |
|  | Number of investigations | Mean rate of missing report | Number of investigations | Mean rate of missing report |
|  |  |  |  |  |
|  |  |  |  |  |
|  |  |  |  |  |

**Q10.2 In your opinion, what are the problems of missing report investigations? (chose all the apply)**

| ① Lack of funding and staff | ② Lack of a stable working system |
| --- | --- |
| ③ Hard to carry out | ④ Poor quality of data |

⑤ Other, please specify: ____________________________________________________________

*Please sort the chosen problems in order of importance：___________________________________*

| **Q11. Does your branch take the responsibility of submitting report of NDRS？** | |
| --- | --- |
| ① Yes, go to Q11.1-11.2 | ② No, ______________(branch name) take the responsibility, go to Q12 |

**Q11.1 Describe the organizations to which reports were submitted. Check  the suitable one.**

| Organization | Report submitted | | Periodicity of report submission | | | | | The number of the reports submitted in last calendar year |
| --- | --- | --- | --- | --- | --- | --- | --- | --- |
|  | Yes | No | Weekly | Monthly | Quarterly | Annually | Other, please specify: |  |
| County health administration department |  |  |  |  |  |  |  |  |
| Prefecture health administration department |  |  |  |  |  |  |  |  |
| Province health administration department |  |  |  |  |  |  |  |  |
| Prefecture-level CDC |  |  |  |  |  |  |  |  |
| Provincial-level CDC |  |  |  |  |  |  |  |  |
| China CDC |  |  |  |  |  |  |  |  |
| MOH |  |  |  |  |  |  |  |  |
| Other, please specify: |  |  |  |  |  |  |  |  |

**Q11.2 Describe the feedbacks received within last calendar year. Check  the suitable one.**

| Organization | Feedback | | Forms of feedback | | | | The number of feedbacks received in last calendar year |
| --- | --- | --- | --- | --- | --- | --- | --- |
|  | Yes | No | Phone call | Official report | Bulletin | Other, please specify: |  |
| County health administration department |  |  |  |  |  |  |  |
| Prefecture health administration department |  |  |  |  |  |  |  |
| Province health administration department |  |  |  |  |  |  |  |
| Prefecture-level CDC |  |  |  |  |  |  |  |
| Provincial-level CDC |  |  |  |  |  |  |  |
| China CDC |  |  |  |  |  |  |  |
| MOH |  |  |  |  |  |  |  |
| Other, please specify: |  |  |  |  |  |  |  |

| **Q12. Have your branch been supervised in last calendar year for NDRS management?** | | |
| --- | --- | --- |
| ① Yes, go to Q12.1-12.3 | ② No, go to Q 13 | ③ I don’t know, go to Q13 |

**Q12.1 Specify the number of supervisions received by your branch within last calendar year.**

| Supervisor | Total number of supervisions | The number of supervisions for | | | |
| --- | --- | --- | --- | --- | --- |
|  |  | Information network maintenance | Data management | Quality of report | Other, please specify: |
| County health administration department |  |  |  |  |  |
| Prefecture health administration department |  |  |  |  |  |
| Province health administration department |  |  |  |  |  |
| Prefecture-level CDC |  |  |  |  |  |
| Provincial-level CDC |  |  |  |  |  |
| China CDC |  |  |  |  |  |
| MOH |  |  |  |  |  |
| Other, please specify: |  |  |  |  |  |

**Q12.2 Do you think the activities of NDRS in your county have been improved by these supervisions or not? Check  the suitable one.**

| Activity | Not at all | Partially | Substantially | Fully or almost fully |
| --- | --- | --- | --- | --- |
| Data collection |  |  |  |  |
| Data analysis |  |  |  |  |
| Policy support |  |  |  |  |
| Funding support |  |  |  |  |

**Q12.3 In your opinion, what are the problems of these supervisions? (chose all the apply)**

| ①They are not productive of any important findings | ②The frequency is too low |
| --- | --- |
| ③ The frequency is too high | ④ Lack of feedback |

⑤ Other, please specify:_____________________________________________________________

*Please sort the chosen problems in order of importance：___________________________________*

| **Q13. Did your branch made supervisory visits in last calendar year?** | | |
| --- | --- | --- |
| ① Yes, go to Q13.1 | ② No, go to Q13.2 | ③ I don’t know, go to Q14 |

**Q13.1 Specify the number of the visits made by your branch within last calendar year.**

| Organization been supervised | Total number of visits | The number of visits for | | | |
| --- | --- | --- | --- | --- | --- |
|  |  | Information network maintenance | Data management | Quality of report | Other, please specify: |
| Hospitals |  |  |  |  |  |
| Health centers |  |  |  |  |  |
| Other medical facilities |  |  |  |  |  |
| Other, please specify: |  |  |  |  |  |

**Q13.2 Describe the most usual reason for not making supervisory visits**

① It is other branch’s responsibility to do the visits. The branch is :_______________________

| ② It is not required | ③ It is not necessary |
| --- | --- |
| ④ Lack of fund | ⑤ Lack of staff |

⑥ Other, please specify:_____________________________________________________________

| **Q14. Have the working staffs in your branch ever been trained on communicable disease surveillance?** | | |
| --- | --- | --- |
| ① Yes, specify how long_________ | ② No, go to Q15 | ③ I don’t know, go to Q15 |

| **Q15. Describe the training course received by the working staffs in your branch within last calendar year.** | | | | | | |
| --- | --- | --- | --- | --- | --- | --- |
| Training | Total trained person-times | Trained person-times of: | | | | |
|  |  | Law and regulation | Network maintenance | Data management and analysis | Epidemiological investigation | Other, please specify: |
| Prefecture-level |  |  |  |  |  |  |
| Province-level |  |  |  |  |  |  |
| National |  |  |  |  |  |  |
| International |  |  |  |  |  |  |

| **Q16. Have your branch provided training courses about communicable disease surveillance in last calendar year?** | | |
| --- | --- | --- |
| ① Yes, go to Q16.1 | ② No, go to Q16.2 | ③ I don’t know, go to Q17 |

**Q16.1** **Describe the trainings courses provided by your branch within last calendar year**.

| Training course | Total number trainings | The number of trained person-times from: | | |
| --- | --- | --- | --- | --- |
|  |  | Hospitals | Health centers | Other |
| Law and regulation |  |  |  |  |
| Network maintenance |  |  |  |  |
| Data management and analysis |  |  |  |  |
| Epidemiological investigation |  |  |  |  |
| Other, please specify: |  |  |  |  |

**Q16.2 Describe the most usual reason for not providing trainings**

① It is other branch’s responsibility to provide trainings. The branch is: ______________________

| ② It is not required | ③ It is not necessary | ④ Lack of fund and equipment |
| --- | --- | --- |
| ⑤ Lack of trainers | ⑥ Lack of training materials | |

⑦ Other, please specify: ____________________________________________________________

| **Q17. List the equipments used for NDRS in your branch.** | | | | | | |
| --- | --- | --- | --- | --- | --- | --- |
| Does your branch have access to internet? ① Yes, it is stable ② Yes, but it is unstable ③ No | | | | | | |
| Equipment | Total  number | Are they working well? | | | Do they need updating? | |
|  |  | Fully or almost fully | Partially | Not at all | Yes | No |
| Computer |  |  |  |  |  |  |
| Laptop |  |  |  |  |  |  |
| Fixed phone |  |  |  |  |  |  |
| Fax |  |  |  |  |  |  |
| Printer |  |  |  |  |  |  |

**Q17.1 Do you think the existing equipment can satisfy the demand of NDRS or not?**

| ① Yes | ② No | ③ I don’t know |
| --- | --- | --- |

| **Q18. Do you know your branch’s NDRS funding source?** | |
| --- | --- |
| ① Yes, go 18.1 | ② No, go to Q19 |

**Q18.1** **Describe your branch’s NDRS funding sources of last calendar year.**

Total fund is ________________________ Yuan RMB

Source 1 _________________________ (title) afforded ______________________ Yuan RMB

Source 2 _________________________ (title) afforded ______________________ Yuan RMB

Source 3 _________________________ (title) afforded ______________________ Yuan RMB

| **Q19. Do you know your branch’s expenditure on NDRS?** | |
| --- | --- |
| ① Yes, go to Q19.1-19.2 | ② No, go to Q20 |

**Q19.1 Specify the percentages of each items of expenditure on NDRS within last calendar year (%).**

| Communication | Equipment | Travel | Training | Office supplies | Allowance | Other |
| --- | --- | --- | --- | --- | --- | --- |
|  |  |  |  |  |  |  |

**Q19.2 Do you think the existing financial support can satisfy the demand of NDRS or not?**

| ① Yes | ② No | ③ I don’t know |
| --- | --- | --- |

| **Q20. Do the working staffs in your branch have overtime pay or paid leave for working for NDRS on weekends or holidays?** | | | |
| --- | --- | --- | --- |
| ① None | ② Paid leave | ③ ___Yuan/day overtime pay | ④ I don’t know |

| **Q21. In your opinion, what are the problems of NDRS in your county?（chose all apply）** | |
| --- | --- |
| ① Insufficient coverage | ② Lack of well-functioned equipment |
| ③ Lack of training staff | ④ Imperfect reporting system |

⑤ Other, please specify:____________________________________________________________

*Please sort the chosen supports in order of importance：___________________________________*

| **Q22. In your opinion, what kind of supports are needed for the NDRS improvement in your county?（chose all apply）** | | |
| --- | --- | --- |
| ① Policy support | ② Financial and equipment support | ③ Integration support |
| ④ Staff training | ⑤ Technique support |  |

⑥ Other, please specify:_____________________________________________________________

*Please sort the chosen supports in order of importance：___________________________________*

| **Q23. What is your suggestion to improve the performance of NDRS in your county？** |
| --- |

________________________________________________________________________________________________________________________________________________________________________________________________________________________________________________

________________________________________________________________________________

**Thank you for your time**

**Please send the finished questionnaire back to...... before../../..**
